# Activation of autophagy and paraptosis in retinal ganglion cells after retinal ischemia and reperfusion injury in rats

**DOI:** 10.3892/etm.2014.2084

**Published:** 2014-11-21

**Authors:** TING WEI, QIANYAN KANG, BO MA, SHAN GAO, XUEYING LI, YONG LIU

**Affiliations:** 1 Department of Ophthalmology, The First Affiliated Hospital, Medical School of Xi’an Jiaotong University, Xi’an, Shaanxi 710061, P.R. China; 2 Institute of Neurobiology, National Key Academic Subject of Physiology, Medical School of Xi’an Jiaotong University, Xi’an, Shaanxi 710061, P.R. China

**Keywords:** autophagy, paraptosis, retinal ischemia/reperfusion injury, retinal ganglion cells

## Abstract

Glaucoma is a neurodegenerative disease characterized by elevated intraocular pressure (IOP), which causes retinal ischemia and progressive neuronal death. Retinal ischemia/reperfusion (RIR) injury is a common clinical condition representing the main cause of irreversible visual field defects in humans. The aim of this study was to investigate whether non-apoptotic forms of programmed cell death (PCD) have an effect on RIR injury in an experimental model that replicates features of acute hypertensive glaucoma and to explore the possible underlying mechanisms. The activation of autophagy was investigated in retinal ganglion cells (RGCs) following RIR in comparison with a control group, using immunofluorescence against microtubule-associated protein 1 light chain 3 (LC3). RIR injury increased LC3 expression in the cytoplasm of RGCs in the ganglion cell layer (GCL) 6 h after the insult, and the increased expression was sustained throughout the experimental period. Following RIR insult, the number of neurons in the GCL significantly decreased. Ultra-structural analyses showed that double- or multiple-membrane autophagosomes were markedly accumulated in the cytoplasm of RGCs following IOP elevation. Since there are no known markers for paraptosis, its identification was based on morphological criteria. Electron microscopy (EM) analysis revealed severe structural alterations associated with cytoplasmatic vacuolization within the 6 h after RIR injury and RGC death. EM also revealed that vacuoles were derived predominantly from the progressive swelling of the endoplasmic reticulum (ER) and/or mitochondria in RGCs after RIR injury. The results provide novel evidence implicating an important role of autophagy and paraptosis in the pathogenesis of RIR injury. Autophagy and paraptosis take place during developmental cell death in the nervous system as well as in certain cases of neurodegeneration. Therefore, targeting autophagy and paraptosis could have therapeutic potential for the prevention of glaucoma involving RIR injury.

## Introduction

Glaucoma is a neurodegenerative disease in which progressive retinal ganglion cells (RGCs) loss and irreversible visual field defects occur. Elevated intraocular pressure (IOP) is considered to be a major causative factor (1). However, in clinical practice, even when the IOP has been well controlled, deterioration of the visual function of a patient may continue to proceed in certain cases. Currently, there are no effective treatments available for the prevention of glaucoma.

Retinal ischemia/reperfusion (RIR) injury is a common clinical condition that is the main cause of loss of vision in humans (2). RIR occurs in a variety of ocular pathologies, including acute glaucoma, diabetic retinopathy and retinal vascular occlusion (2,3). An animal model of RIR injury, which mimics the clinical situation of acute glaucoma, is frequently used to study RGC loss or dysfunction following ischemic injury (4). However, the mechanism of RGC death in glaucoma is not fully understood. Despite the efforts that have been made to understand the pathological processes responsible for the insults to the retina, it is currently not possible to prevent this complication.

Autophagy is an evolutionarily conserved process that leads to the degradation of long-lived proteins and recycling of cellular constituents (5). According to its mechanism and function, autophagy can be divided into three pathways in all eukaryotic cells: macroautophagy, microautophagy and chaperone-mediated autophagy (CMA) (6). Basal autophagy is crucial, particularly in neurons. It occurs in various organs and assists in maintaining homeostatic function during protein and organelle turnover (6,7). Under various pathophysiological forms of stress, autophagy can be induced to meet the needs of developmentally related structural remodeling, and to clear toxic or misfolded proteins, superfluous or damaged organelles and invading microorganisms (8,9). During autophagy, proteins and organelles are degraded and form double- or multiple-membrane autophagic vesicles, the autophagosomes, which subsequently fuse with the lysosomal compartment to form autolysosomes (10). In some cells, the excessive accumulation of autophagosomes and autophagolysosomes leads to cell death, which is known as autophagic cell death or type II programmed cell death (PCD) (7). Autophagic death is activated in the pathophysiology in a variety of disorders, particularly in neuronal cell death associated with chronic neurodegenerative disorders and acute ischemia/hypoxia neuronal injury (11). Despite recent studies which have shown that autophagy is activated in RGCs following optic nerve transection and glaucoma, autophagic processes in RGC death remain poorly understood (12,13).

Although apoptosis is sometimes equated with PCD, it has become evident that several non-apoptotic forms of PCD, including autophagic cell death and cytoplasmic cell death exist (14). A type of PCD, dubbed paraptosis, that often exists in parallel with apoptosis was originally identified in 2000 (15). Paraptosis is characterized by extensive cytoplasmic vacuolization without nuclear fragmentation that begins with progressive swelling of the endoplasmic reticulum (ER) and/or mitochondria. Apoptotic morphology and DNA fragmentation are absent. Paraptosis typically does not respond to caspase inhibitors (z-VAD-fmk, boc-aspartate fluoromethylketone, p35 and XIAP) or Bcl-XL; nor does it involve activation of poly(ADP-ribose) polymerase (PARP) cleavage and chromatin condensation (16). Paraptosis can be triggered by the TNF receptor family member TAJ/TROY, the apoptotic protein Bax, and human insulin-like growth factor I receptor (17). However, due in part to the lack of specific markers, paraptosis has not been thoroughly investigated and may thus have been underestimated. Paraptosis can take place in certain pathological conditions, such as excitotoxicity, ischemia and neurodegeneration. In neurodegenerative diseases, such as Huntington’s disease and amyotrophic lateral sclerosis, not all neuronal cell death appears to occur via apoptosis (18,19). Paraptosis has begun to garner attention as a significant contributor to ischemia-associated damage (12). There are, however, few reports on retinal insult through mechanisms involving paraptosis (20).

The aim of this study was to investigate the involvement of various cell death mechanisms in a model of RIR injury induced by acute IOP increase. Changes of the features of autophagy and paraptosis were investigated at various time points following RIR injury.

## Materials and methods

### Experimental animals

A total of 48 adult male Sprague-Dawley rats weighing 220–250 g were provided by the Experimental Animal Center of the Medical School of Xi’an Jiaotong University (Xi’an, China). They were housed in individually ventilated cages containing wood shavings and maintained in a temperature-controlled environment with free access to food and water and a 12-h light-dark cycle. All experiments were performed in accordance with the Statement on the Use of Animals of the Association for Research in Vision and Ophthalmology, and were approved by the ethics committee of Xi’an Jiaotong University (Xi’an, China).

### Induction of ischemia and reperfusion

Rats were deeply anesthetized by intraperitoneal injection of 10% chloral hydrate (40 mg/kg). Topical analgesia was achieved using 0.4% oxybuprocaine hydrochloride eye drops (Santen, Osaka, Japan). Pupils were dilated with 1% tropicamide eye drops (Santen). A 30-gauge infusion needle, connected to a pressure device, was inserted into the anterior chamber of the right eye. The IOP was elevated to 110 mmHg for 60 min. The cannulating needle was removed after 60 min of retinal ischemic and the IOP was normalized. For each animal, the left eye served as a non-ischemic control. At 6, 12, 24 h, 3 days and 7 days after ischemia, rats were sacrificed by an intraperitoneal overdose injection of chloral hydrate. The right eyes were rapidly enucleated and cut through the pars plana, anterior segment and the vitreous was removed. The harvested retina was immersed in liquid nitrogen prior to analysis. Of the 8 rats sacrificed following ischemia, the control and model eyes of three rats were used for analysis by transmission electron microscopy (TEM) and the eyes from the other five rats were used for cryosections.

### Immunohistochemical staining

For histological examination, the fixed retinas were cut in 12-μm sections using a cryostat microtome. After preincubating with 10% goat serum (Boster Biological Technology Co., Ltd., Wuhan, China) for 30 min, sections were incubated overnight at 4°C with a primary antibody against microtubule-associated protein 1 light chain 3 (polyclonal; anti-rat LC3; rabbit IgG, 1:500; MBL International Corporation, Des Plaines, IL, USA), followed by incubation with fluorescently labeled secondary antibody [fluorescein isothiocyanate (FITC) anti-mouse IgG, 1:200; Beijing CoWin Biosciences Co., Ltd., Beijing, China] for 2 h at room temperature. The sections were counterstained for 10 min with 0.1 mg/ml DAPI (Roche Applied Science, Mannheim, Germany) and rinsed with phosphate-buffered saline. Sections were observed under a fluorescence microscope (Olympus BX51; Olympus Corporation, Tokyo, Japan). DAPI-positive cell were counted at ×400 magnification and images were captured in 6 different areas per retinal with ~442 μm retinal length per area. DAPI-positive cells were counted using Image Pro Plus 6.0 software (Media Cybernetics, Inc. Rockville, MD, USA) and 4–6 samples per group were used for this analysis.

### TEM

Tissue samples were obtained from the three pairs of control and model eyes and 10 fields of each eye were examined by TEM (H-7650; Hitachi, Tokyo, Japan). Specimens were post-fixed with 4% glutaraldehyde in 0.1 mmol/l phosphate buffer (pH 7.4) for 2–4 h and then with 1% osmium tetroxide in 0.1 mmol/l phosphate buffer for 2 h. After dehydrating in applied acetone, the retinal sections were embedded in pure acetone medium. Ultrathin sections (80 nm) were sliced and stained with uranyl acetate and lead citrate (Ted Pella, Inc., Redding, CA, USA). Photographs were taken with a digital CCD camera (Olympus-SIS Veleta, Münster, Germany). Image Pro Plus 6.0 (Media Cybernetics, Inc.) and Photoshop (Adobe, San Jose, CA, USA) software were used to further quantify the TEM images.

### Statistical analysis

Statistical analysis was performed using SPSS statistical software version 13.0 (SPSS, Inc., Chicago, IL, USA). The results are expressed as mean ± standard deviation (SD). One-way analysis of variance (ANOVA) with subsequent post hoc tests was used to compare differences among groups. A P-value <0.05 was considered to indicate a statistically significant difference.

## Results

### Ultrastructural features of autophagy following RIR injury

**Figure 1:**
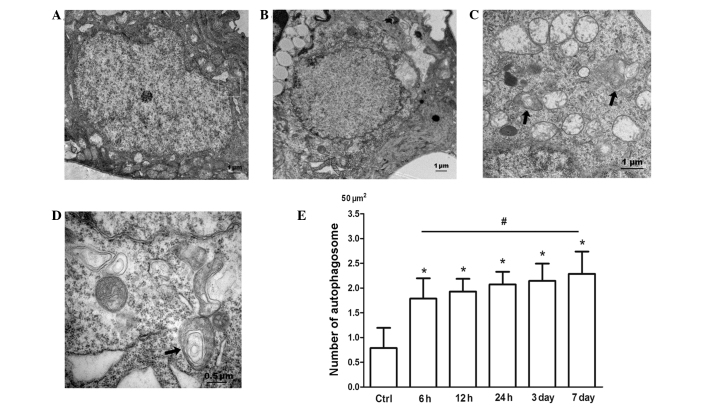
Transmission electron micrograph images of RGCs in the GCL. (A–D) Ultrastructural images of RGCs in the GCL. (A) In a control rat retina, autophagic vesicles are seldom observed. (B and D) The presence of double- or multiple-membrane autophagosomes containing cell organelles (arrows) in the cytoplasm of an experimental rat retina at 7 days after RIR injury and (C) 6 h after RIR injury. (E) Comparison of the numbers of autophagosomes per TEM image (50 mm^2^) in the rat retina. Three eyes were used in each experimental period. Bar represents mean ± SD ^*^P<0.05 compared with the control. RGCs, retinal ganglion cells; GCL, ganglion cell layer; RIR, retinal ischemia/reperfusion.

Morphometric analysis by TEM cell imaging clearly confirmed whether autophagic activity was altered in RGCs following RIR at different time-points (6, 12, 24 h, 3 days and 7 days). The TEM manifestation of double-membrane vesicles surrounding cytoplasmic structures remains the gold standard for identifying autophagosomes (8). Double- and multiple-membrane vacuoles surrounding electron-dense and compacted amorphous contents or whorls of membranous material are properties of autophagosomes (8). Prior to transient ischemia, autophagic events were occasionally observed in the cytoplasm of RGCs in the ganglion cell layer (GCL; Fig. 1A). However, autophagosomes were readily detectable in the cytoplasm of RGCs in the GCL 6 h after RIR injury (Fig. 1C) and were sustained throughout the experimental period. The average number of autophagic vacuoles in the cytoplasm of RGCs was ~0.79/50 μm^2^ prior to IOP elevation. However, RIR insult markedly increased the number of autophagic vacuoles in RGCs in the GCL (Fig. 1B–D). The average number of autophagosomes was a maximum at 7 days after reperfusion at ~2.29/50 μm^2^ (Fig. 1E). Autophagic activities were exhibited as an autophagosome enclosing a damaged mitochondrion, and an autophagic vacuole surrounding partially degraded membranous material. These observations indicate that stimulated autophagic flux in the cytoplasm of RGCs in the GCL is involved in the pathogenesis of RIR injury.

### Immunolocalization of LC3 following IOP elevation

Using immunofluorescence against LC3, a specific form of autophagic protein, the activation of autophagy in RGCs following RIR was analyzed by comparison with the control group. Relatively weak LC3 (green) staining was present in the GCL and inner plexiform layer (IPL). The negative control for immunofluorescence, in which the LC3 primary antibody was omitted, showed no detectable staining. Cell nuclei were counterstained with DAPI. LC3 immunoreactivity, which was observed as clusters of small, intensely stained granules, significantly increased in the GCL and IPL 6 h after the RIR insult. After 6 h of RIR injury, LC3 expression was enhanced in the cytoplasm of RGCs in the GCL, and sustained throughout the experimental period. LC3 immunoreactivity decreased in the IPL 3 days after reperfusion. DAPI-positive cells in the GCL were counted under ×400 magnification. The average number of DAPI-positive cells in the GCL was 26.2±1.92 cells in the control, and 7 days after RIR insult, it was decreased to 16.4±1.67 cells. The reduction in the number of DAPI-positive cells indicated that RIR injury led to extensive loss of neurons in the GCL. The thickness of the IPL and inner nuclear layer (INL) at 7 days after RIR injury also markedly decreased, reflecting the destruction of inner retinal elements. These results indicate that IOP elevation induced the activation of autophagy. In addition, the activation of autophagy apparently increased in the cytoplasm of RGCs 7 days after RIR insult, which coincided with the period of RGC death.

### Ultra-structural features of paraptosis-like cell death induced by RIR injury

**Figure 2:**
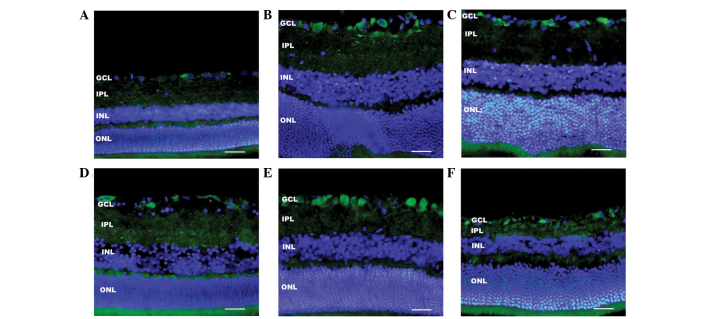
Immunofluorescence showed the time course of LC3 (green) expression in all retina layers after RIR. (A) Control shows relatively weak staining of LC3 in the IPL and GCL. After RIR insult, LC3 immunoreactivity gradually increases in the IPL, and more intense immunoreactivity is observed in the GCL at (B) 6 h, (C) 12 h and (D) 24 h. (E) Three days after RIR, LC3 immunoreactivity decreases markedly in the IPL. (F) LC3 expression is enhanced in the cytoplasm of RGCs in the GCL until 7 days after RIR. Cell nuclei were counterstained with DAPI. The thickness of IPL and INL at 7 days after RIR injury also markedly decreased. Three eyes were used in each experimental period. Scale bars, 50 mm. RIR, retinal ischemia/reperfusion; IPL, inner plexiform layer; GCL, ganglion cell layer; INL, inner nuclear layer; ONL, outer nuclear layer.

**Figure 3:**
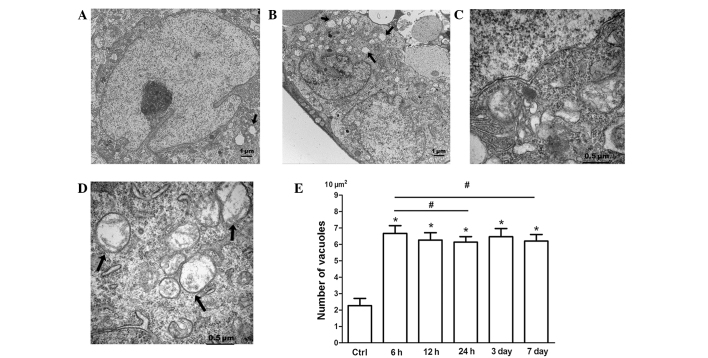
Transmission electron micrograph images of RGCs in the GCL. (A–D) Ultrastructural images of RGCs in the GCL. (A) In the control rat retina, intracellular vacuoles are occasionally observed. (B) Cytoplasmic vacuoles (arrows) are increased in the experimental rat retina at 6 h after RIR injury, and are observed in the absence of cytoplasmic material. (C) A higher magnification image shows intracellular vacuoles were occasionally observed in the retina of the control rat (D) A higher magnification image shows dilated mitochondria and endoplasmic reticulum (arrows). (E) Comparison of the numbers of cytoplasmic vacuoles per TEM image (10 mm^2^) in the rat retina. Three eyes were used in each experimental period. Bar represents mean ± SD ^*^P<0.05 compared with the control. RGCs, retinal ganglion cells; GCL, ganglion cell layer; RIR, retinal ischemia/reperfusion.

Following RIR injury, extensive cytoplasmic vacuolization in RGCs was significant at different end-points (6 h, 12 h, 24 h, 3 days and 7 days). In the control group, intracellular vacuoles surrounding the cell nucleus were occasionally observed (Fig. 2A). Cytoplasmic vacuolization, in which the vacuoles appear clear with no cytoplasmic material in the vacuoles, is a typical feature of paraptosis (15). The present TEM study verified that cytoplasmic vacuolation was evident at 6 h after RIR injury; however, it was accompanied by the simultaneous occurrence of a necrotic-like morphology, autophagy and apoptosis, and therefore showed specific characteristics. When the reperfusion time was increased, RGCs with cytoplasmic vacuolization were more pronounced and extensive cytoplasmic vacuolation occurred at 6 h (Fig. 2B). As illustrated in Fig. 2D, RIR injury caused progressive swelling of the ER and/or mitochondria of RGCs, and the cristae of the mitochondria appeared diffuse. As shown in Fig. 2E, RIR injury significantly increased the number of cytoplasmic vacuoles among the RGCs. Cytoplasmic vesicles were occasionally observed in the RGCs (Fig. 3A and C). The average number of cytoplasmic vacuoles in the RGCs was ~2.27/10 mm^2^ prior to IOP elevation. However, following RIR insult, the number of RGCs displaying cytoplasmic vesicles in the GCL markedly increased. The peak average number of cytoplasmic vesicles appeared at 6 h after IOP elevation, and was ~6.7/10 mm^2^ (Fig. 3E). Increased numbers of cytosolic vesicles remained in the RGCs in the GCL until 7 days after RIR injury. These observations correspond to the pattern of paraptosis in which paraptotic cells are characterized by physical enlargement of the mitochondria and ER (15). Therefore, RIR injury induced RGC death in part via paraptosis. The appearance of swollen organelles may suggest disruption of intracellular homoeostasis due to RIR injury.

## Discussion

Results from the present study indicate that the activation of autophagy and paraptosis is associated with retinal damage caused by RIR insult in an acute hypertensive glaucoma model. In comparison to the control group, there was evidence that autophagy markedly increased in the retina following RIR injury, as demonstrated by changes in the immunohistological staining and changes observed by TEM. Following RIR injury, the accumulation of cytoplasmic vacuoles was observed in the RGCs, suggesting that paraptosis, a non-apoptotic form of PCD, was associated with RIR injury. The occurrence of paraptosis is further supported by the observation of mitochondrial and ER swelling.

Glaucoma is a leading cause of blindness globally. It is a neurodegenerative disease of the optic nerve that can ultimately lead to irreversible damage to visual function (1,2). A transient increase in the IOP threshold can trigger a cascade of damage to the RGCs. Even when pathological IOP is lowered to the normal range, neural damage in the retina may continue to proceed. A number of studies demonstrate that all kinds of PCD, including apoptosis and autophagy, play important roles in the glaucomatous retina of glaucoma patients and mammalian models (12,14,20). Methods aimed at improving the understanding of molecular mechanisms within each PCD and blocking the PCD may be helpful for neuronal survival and preservation of visual function. The use of animal models of RIR injury induced by the transient elevation of IOP, mimicking clinical situations of acute angle-closure glaucoma, may increase the knowledge of the mechanisms underlying the retinal neuronal death of glaucoma and thus may provide new insights into the disease therapy.

The present study showed that in the animal model, the involvement of autophagy in the neurodegenerative processes accompanying ischemia in the GCL was sustained following acute IOP elevation. Immunostaining analysis demonstrated that the expression of LC3 in the GCL increased 6 h after IOP elevation. This preceded significant RGC loss, and was maintained throughout the experimental period. However, following a period of significant RGCs loss at ~7 days after IOP induction, LC3 immunoreactivity in the cytoplasm of RGCs in the GCL markedly increased. However, an increase of LC3 expression does not appear to be specific for autophagy. In addition to its important role in autophagy, LC3 has been reported to be involved in non-autophagic cytoplasmic vacuolization (21). Moreover, previous studies have indicated a significant reduction in retinal thickness at 7 days after RIR (12,13). Consistent with these studies, the ocular hypertension model in the present study also exhibited a marked reduction in retinal thickness at 7 days after RIR injury, reflecting the destruction of the inner retinal elements. Ultrastructural features of double- or multiple-membrane acidic autophagic vesicles were observed by TEM in the RGCs until 7 days after IOP elevation. This increase of autophagy in the glaucomatous retina may act to recycle damaged material and lead to cell death. Depending on the cellular milieu, autophagy, as a lysosome-mediated self-degradation process of eukaryotic cells, can either promote survival or act as an alternative mechanism of PCD for neurons (6,22). Autophagy, as a defense mechanism for the removal of toxic multimeric complexes and aggregated proteins in neurodegenerative diseases, may also occur for self-digestion and self-clearance and the suppression of basal autophagy may cause neurodegeneration (6,8). By contrast, autophagy can promote cell death through excessive cellular autolysis, and by degrading fundamental cellular constituents. Autophagic markers such as LC3 participate in various steps of cerebral focal ischemia following the insult (23).

Paraptosis is a recently defined type of PCD and little is known about its mechanism. There is a continuing effort to identify paraptosis-specific changes, and only in the last few years has the first proteomic analysis of this type of non-apoptotic PCD been described (16). In the present study, evaluation by TEM confirmed that RIR insult induced irregular cytoplasmic vacuolization in RGCs. Further characterization of the cytoplasmic vacuolization indicated that it was associated with ER and/or mitochondria dilation along with preservation of nuclear chromatin. These results indicate that the cytoplasmic vacuolization induced by RIR insult might be associated with paraptosis, and is suggestive of paraptotic cell death. Swelling of the ER and mitochondria has been demonstrated to be associated with a disruption of intracellular homoeostasis (24). Furthermore, in a previous study it was found that autophagy inhibitors did not prevent but, on the contrary, enhanced the formation of cytoplasmic vacuolization (25). Paraptosis occurs during cell differentiation as the nervous system develops, as well as in many neurodegenerative diseases (16). The association between paraptosis and neurodegeneration has been demonstrated by several studies highlighting the fact that neurons are vulnerable to stress-related signals involved in the paraptosis process (15,26). Further studies of paraptosis are required to elucidate the mechanism of PCD and may facilitate the identification of future therapeutic approaches for ischemia. Therapies for the induction of non-apoptotic forms of PCD such as paraptosis might suppress the multi-drug resistant phenotype often concerned with resistance to apoptosis (27). Thus, methods of targeting PCD may be beneficial as alternative to apoptosis-based therapeutics.

The results of the present study indicated that paraptosis and autophagy occurred simultaneously in the RIR-insulted RGCs. All three types of PCD, namely paraptosis, autophagy and apoptosis, can be induced by RIR insult in RGCs. In a previous study, Kim and Park found that acute IOP elevation was associated with a variety of changes in cell death and cell survival pathways in RGCs (28). Although paraptosis and autophagy were induced in RIR-insulted RGCs, they may not have contributed to cell death; they may have served as a mechanism of cell survival. The preliminary observations in this study focus on the high incidence of autophagy and paraptosis in the GCL where RGCs die by this nonapoptotic pathway. The roles of individual types of cell death and the coexistence of multiple cell death types (paraptosis, autophagy and apoptosis) have been reported in recent studies concerning ischemic injury, neurodegeneration and viral infection (25,27,29,30). For the identification of the type of cell death, and even for the quantification of certain processes, EM remains one of the most accurate methods; for example, paraptosis was first discovered by TEM (15). In addition, EM is able to demonstrate the basic characteristics of cell death and unexpected associations of subcellular features in the same cell, such as apoptotic, paraptotic and autoschizic changes, which may suggest a kind of cell reprogramming. Ultrastructural features of RGCs that die in the retina are useful in making a clear morphological comparison between different activated PCD programs in a variety of clinical statuses, not only for diagnosis and prognosis, but also to establish appropriate therapeutic interventions. The identification of biochemical markers of PCD and other methodological improvements should increase our understanding of the multiple roles of cell death programs in neuronal fate.

In summary, the results of the present study confirm previous reports that apoptosis produces retinal cell death after RIR injury, and also demonstrate that dysregulated autophagy and paraptosis may participate in the death of RGCs under transiently elevated IOP. An emerging consensus is that autophagy and paraptosis have dual roles, acting as a prosurvival mechanism or as a process of progressive deterioration leading to cell death. Numerous microenvironmental factors may induce and/or activate one or more biochemical pathways of autophagy and paraptosis. These molecular events can be visualized by TEM at the cellular level, and the type of death can be characterized morphologically. Therefore, these observations enhance our understanding of the mechanism of non-apoptotic cell death in the retina following RIR injury. Targeting autophagy and paraptosis, either by inhibition or by enhancement, could represent a potential approach for new and supporting therapeutic interventions for diseases of the nervous system, in retinal ischemia as well as for other neurodegenerative diseases.
